# Global, regional, and national burden of age-related macular degeneration, 1990–2019: an age-period-cohort analysis based on the Global Burden of Disease 2019 Study

**DOI:** 10.3389/fpubh.2024.1486168

**Published:** 2024-10-22

**Authors:** Yufan Huang, Tong Tang, Dongyue Wang, Yunxia Gao, Ming Zhang

**Affiliations:** ^1^Department of Ophthalmology, West China Hospital, Sichuan University, Chengdu, China; ^2^Institutes for Systems Genetics, Frontiers Science Centre for Disease-related Molecular Network, West China Hospital, Sichuan University, Chengdu, China

**Keywords:** macular degeneration, Global Burden of Disease, disability-adjusted life years, age-period-cohort, disparities

## Abstract

**Objectives:**

This study aimed to explore the burden of disease and disparities in age-related macular degeneration (AMD) at the global, regional, and national levels from 1990 to 2019 using data from the Global Burden of Disease (GBD) 2019 study, with a particular focus on associations with age, period, and cohort.

**Methods:**

We derived disability-adjusted life years (DALYs) and age-standardized rates of AMD from the GBD 2019. We used an age-period-cohort (APC) model to estimate the overall annual percentage changes in DALYs (net drifts), the annual percentage changes in different age groups (local drifts), the longitudinal age profiles (longitudinal age-specific rates), and the relative risks of period and cohort (period and cohort effects) between 1990 and 2019. Further analysis was conducted by country, region, gender and sociodemographic index (SDI).

**Results:**

Globally, the number of DALYs increased from 296771.9321 (95% uncertainty interval [UI], 205462.8041–418699.8184) in 1990 to 564055.0967 (95% UI, 392930.6967–789194.6407) in 2019 (59.7% were female), while the age-standardized DALYs rates decreased from 8.29 per 100,000 (95% UI, 5.8–11.58/100,000) to 7.05 per 100,000 (95% UI, 4.92–9.84/100,000). With increasing age, the burden of AMD increased, and the DALYs rates in female was greater than that in male in all age groups. The burden of disease varied across SDI regions and countries. The top three countries in terms of the number of DALYs were China, India and Italy, accounting for 45% of the global total.

**Conclusion:**

The burden of AMD varied according to SDI, country, and sex from 1990 to 2019. Due to global population growth and aging, AMD will continue to be a major public health problem in the future, and relevant health policies need to be continuously improved and optimized.

## Introduction

Age–related macular degeneration (AMD) is a degenerative disease of the macula. Globally, AMD is one of the leading causes of severe and irreversible loss of vision, accounting for 8.7% of the world’s total blindness, just behind cataract and uncorrected refractive error (1–3). Loss of vision caused by AMD seriously affects quality of life and increases psychological and nursing burdens (4, 5). The abnormal expression of vascular endothelial growth factor (VEGF) plays a crucial role in the pathophysiological process of AMD (6–8). Although anti-VEGF agents can effectively alleviate disease progression, the prevalence and burden of AMD will become more severe public health problems due to the increasing life expectancy of the global population, aging population, and declining mortality in most countries and regions. Wong et al. showed that the number of people suffering from AMD is expected to increase from 196 million in 2020 to 288 million in 2040 (9).

There have been a number of epidemiological studies on AMD, including the Blue Mountains Eye Study, the Singapore Epidemiology of Eye Disease Study, and the Los Angeles Latino Eye Study (10–12). Many previous studies have analysed the prevalence and burden of AMD in different countries, sexes, and age groups (13, 14). However, these findings cannot be generalized to other parts of the world, and few studies have analysed the burden of disease and differences in AMD globally.

The Global Burden of Disease 2019 Study (GBD 2019) aims to systematically analyse the burden of various human diseases, including AMD, from 1990 to 2019 and discuss trends in their prevalence, incidence, and disability-adjusted life years. The GBD 2019 can identify the areas with the greatest burden and provide some reference information about public health policy and resource allocation. Understanding the global epidemiological changes in AMD is critical for improving prevention and treatment strategies. Compared to traditional epidemiological metrics, the burden of disease in AMD is often measured by using disability-adjusted life years (DALYs), which can quantify the health costs of disease. DALYs refer to the total number of healthy life years lost from onset to death, which is the sum of the years of life lost due to disease-related premature death and the years lived with disability due to disease (15). Moreover, the epidemiological trends of a disease are the result of interactions among age, period, and cohort. Analysing the relationship between the burden of disease and age, period, and cohort can also assess the effectiveness of health care services in all aspects while identifying shortcomings and areas for improvement in current treatment. However, previous AMD-related epidemiological studies have not evaluated the impact of age, period, or cohort on the burden of disease in AMD. In addition, since the relationships among age, period, and cohort are perfectly linear (birth cohort = period − age), this inherent correlation makes it extremely difficult to statistically directly estimate their independent effects on a disease, which is known as the identification problem (16). The age-period-cohort (APC) model used in this study can indirectly solve this problem by producing estimable APC parameters and functions without imposing any subjective or arbitrary constraints on the model parameters (17). The APC model reveals the contributions of age-related biological factors, time-related medical technologies, and social factors to disease trends. It is an advanced approach that goes beyond traditional analysis in health and social sciences (16).

Therefore, this study mainly used the APC model to analyse the global and regional trends in AMD DALYs from 1990 to 2019. The significance of this study is to inform prevention and treatment strategies, reduce the impact of AMD on human health, and improve quality of life worldwide.

## Methods

### Data sources

The GBD 2019 provides the most up-to-date descriptive epidemiological data on a total of 369 diseases and injuries for 204 countries and territories from 1990 to 2019 (15). A more detailed introduction to the GBD 2019 study and how to use it have been comprehensively described in previous studies (15, 18). All data in this study were obtained from GBD 2019. DALYs are used to express the burden of disease. The DALYs rate is adjusted to the number of DALYs per 100,000 population based on population size, while the age-standardized DALYs rate is further adjusted according to the age structure. Each indicator from the GBD 2019 database has a 95% uncertainty interval (UI) to measure the uncertainty associated with medical statistics or parameter estimates (15, 18).

The sociodemographic index (SDI) is a quantitative measure of the sociodemographic development of a country or region. The SDI is an indicator estimated as a composite of *per capita* income, average years of education, and the fertility rate among female under 25 years of age. It ranges from 0 to 1, with higher values indicating higher socioeconomic levels (15). The SDI is divided into 5 groups: low SDI, low-middle SDI, middle SDI, high-middle SDI, and high SDI.

### APC model analysis

In this study, the APC model framework was used to estimate temporal trends in the burden of disease by age, period, and birth cohort. Age effect is an important factor in the occurrence and development of age-related diseases. The period effect refers to the change in a series of human factors that affect the incidence of disease in a population, including the innovation of disease diagnosis technology, improvement of screening and early detection mechanisms, updating of disease definitions and registration standards, and continuous improvement of treatment methods. The cohort effect refers to the change in the incidence of disease due to the different levels of exposure to risk factors faced by different birth cohorts over the life course. When constructing the APC model, the estimates of AMD DALYs and population data of each country or region obtained in the GBD 2019 were used as input data. For consistency in the analysis of the age and time dimensions, the age and period intervals usually must be equal. Therefore, this study used ten age groups (from 50–54 to 95+ in five-year age group intervals) and divided the entire study period (1990–2019) into six five-year periods (from 1990–1994 to 2015–2019 in five-year intervals) and partially overlapping ten-year birth cohorts. This study used the APC model to fit the overall trend of DALYs over time, expressed as the annual percentage change in DALYs (i.e., the net drift, % per year), which contains the trend of DALYs change due to calendar time (i.e., the period effect) and different birth cohorts (i.e., the cohort effect). At the same time, this study also performed an in-depth analysis of the temporal trend of DALYs in each age group, which was quantified by the annual percentage change of DALYs in specific age groups (i.e., local drift, % per year), which can reflect cohort effects (19). Typically, when the drift reaches a level of ±1% per year or more, we consider this to be a material change (19). Moreover, the age effect of the APC model output is expressed by the fitted longitudinal age-specific rates, while the period or cohort effect is measured by calculating the relative risk (the choice of reference period or cohort is arbitrary and does not affect the interpretation of the results) (19). The statistical tests were two-sided, and *p* < 0.05 was considered significant. All data analysis and visualization were carried out in R software (v4.3.1).

### Ethical considerations

This study used data from the GBD 2019 Study, which was approved by the institutional review board of the University of Washington. Original data were collected with informed consent from the study participants or with a waiver from the institutional review board. As this was a secondary analysis of publicly available data, no further review by an institutional review board was required following the data use agreement of The Institute for Health Metrics and Evaluation.

## Results

### Global and regional trends in AMD DALYs, 1990–2019

The global burden of AMD is shown in Table 1. Globally, the number of DALYs in AMD has increased by approximately 90%. In 2019, among the different SDIs, the middle-SDI region had the highest number of DALYs. This region was followed by the high-middle SDI region, and the low SDI region had the lowest number of DALYs. To eliminate the influence of age and population composition, the age-standardized DALYs rate (per 100,000) was used to further assess the burden of disease (Table 1). The global burden of AMD has declined by approximately 15%. The age-standardized DALYs rates of AMD in different SDIs also showed a downwards trend. In 2019, the age-standardized DALYs rate was lowest in the high-SDI region. Compared to other SDIs, the age-standardized DALYs rate was highest in the low-SDI region, which was contrary to the number of DALYs. These results indicate that the age-standardized DALYs rate is more suitable than the number of DALYs for reflecting the true burden of AMD in low-SDI regions.

**Table 1:** Trends in AMD DALYs globally and across sociodemographic index quintiles, 1990–2019.

	1990		2019		1990–2019
	DALYs number ×1,000 (95% UI)	Age-standardized DALYs rate per 100,000 (95% UI)	DALYs number ×1,000 (95% UI)	Age-standardized DALYs rate per 100,000 (95% UI)	APC model estimates net drift of DALYs, % per year (95% CI)
Global	296.77(205.46–418.7)	8.29(5.8–11.58)	564.06(392.93–789.19)	7.05(4.92–9.84)	−0.57(−0.72 to −0.43)
Sociodemographic index					
Low SDI	23(16.02–32.35)	11.38(7.98–15.91)	46.04(31.7–64.13)	10.29(7.06–14.36)	−0.37(−0.43 to −0.3)
Low-middle SDI	56.34(38.63–79.39)	10.71(7.4–15.07)	98.28(67.79–136.49)	7.78(5.42–10.82)	−1.13(−1.23 to −1.02)
Middle SDI	80.17(54.57–113.96)	8.93(6.14–12.58)	178.88(123.12–252.64)	7.62(5.26–10.74)	−0.5(−0.83 to −0.16)
High-middle SDI	80.58(55.73–112.86)	8.42(5.92–11.74)	151.43(105.8–210.71)	7.47(5.24–10.37)	−0.42(−0.56 to −0.29)
High SDI	56.55(38.7–79.75)	5.36(3.65–7.52)	89.19(61.51–125.37)	4.23(2.91–5.94)	−0.81(−0.83 to −0.79)

The age-standardized DALYs rate was calculated using direct standardization of the GBD 2019 Global Standard population. The parentheses after the GBD estimate indicate 95% uncertainty intervals. The net drift is an estimate obtained from the APC model that represents the overall annual percentage change in DALYs, with 95% confidence intervals in parentheses. AMD, age-related macular degeneration; DALYs, disability-adjusted life years; SDI, sociodemographic index; APC, age-period-cohort.

### National trends in AMD DALYs, 1990–2019

From 1990 to 2019, AMD DALYs varied significantly among different countries or regions. Among the 204 countries and territories, 12 countries had more than 10,000 AMD DALYs in 2019, of which China (142.07, 95% UI: 97.1–197.28), India (86.26, 95% UI: 59.29–122.4) and Italy (25.59, 95% UI: 17.49–36.52) accounted for 45% of the global AMD DALYs. Although the number of AMD DALYs in China and India increased significantly, the age-standardized DALYs rates decreased by 3.6 and 35%, respectively. In China, the net drift change was relatively stable (−0.16, 95% confidence intervals [CI]: −0.53 to 0.2). In India, the net drift change was more significant (−1.5, 95% CI: −1.63 to −1.37) (Supplementary Table S1).

**Figure 1:**
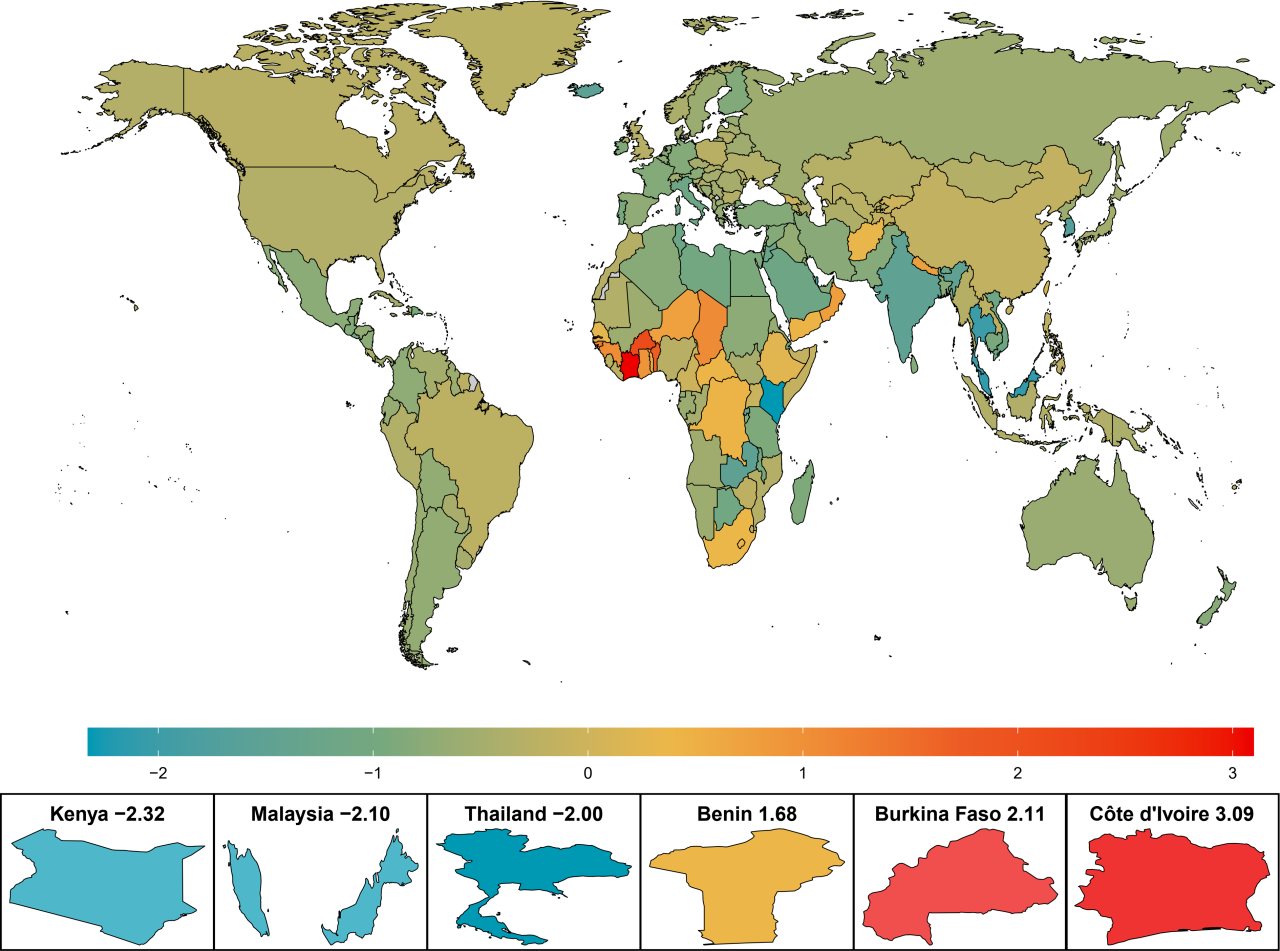
Net drift of AMD DALYs in 204 countries and regions from 1990 to 2019. The color heatmap transitions from blue on the left to red on the right, indicating a gradual increase in the net drift of AMD DALYs, which also signifies that the burden of disease faces greater challenges. AMD, age-related macular degeneration; DALYs, disability-adjusted life years.

The net drift of AMD DALYs in different countries or regions is shown in Figure 1. Among the 204 countries and regions, the number of DALYs in 20 countries showed an upwards trend (net drift>0), mainly in Africa (75%, n/n = 15/20). Among them, Côte d’Ivoire, a West African country, had the greatest increase in DALYs, with a net drift of 3.09% (95% CI: 2.97–3.21), followed by Burkina Faso (2.11, 95% CI: 2.01–2.21), Benin (1.68, 95% CI: 1.6–1.75), the Republic of Nea-Bissau (1.28, 95% CI: 1.18–1.39), Gambia (1.24, 95% CI: 1.18–1.29), Chad (1.06, 95% CI: 1.04–1.08), and Guinea (1.05, 95% CI: 1.01–1.1) (Supplementary Table S1). Kenya, an African country, experienced a 49.8% decrease in the age-standardized DALYs rate and the largest change in net DALYs. The change in the burden of AMD in two Asian countries, Malaysia and Thailand, is also noteworthy, and the net drift of DALYs in both countries also decreased significantly.

### Temporal trends in the AMD DALYs rate across different age groups

**Figure 2:**
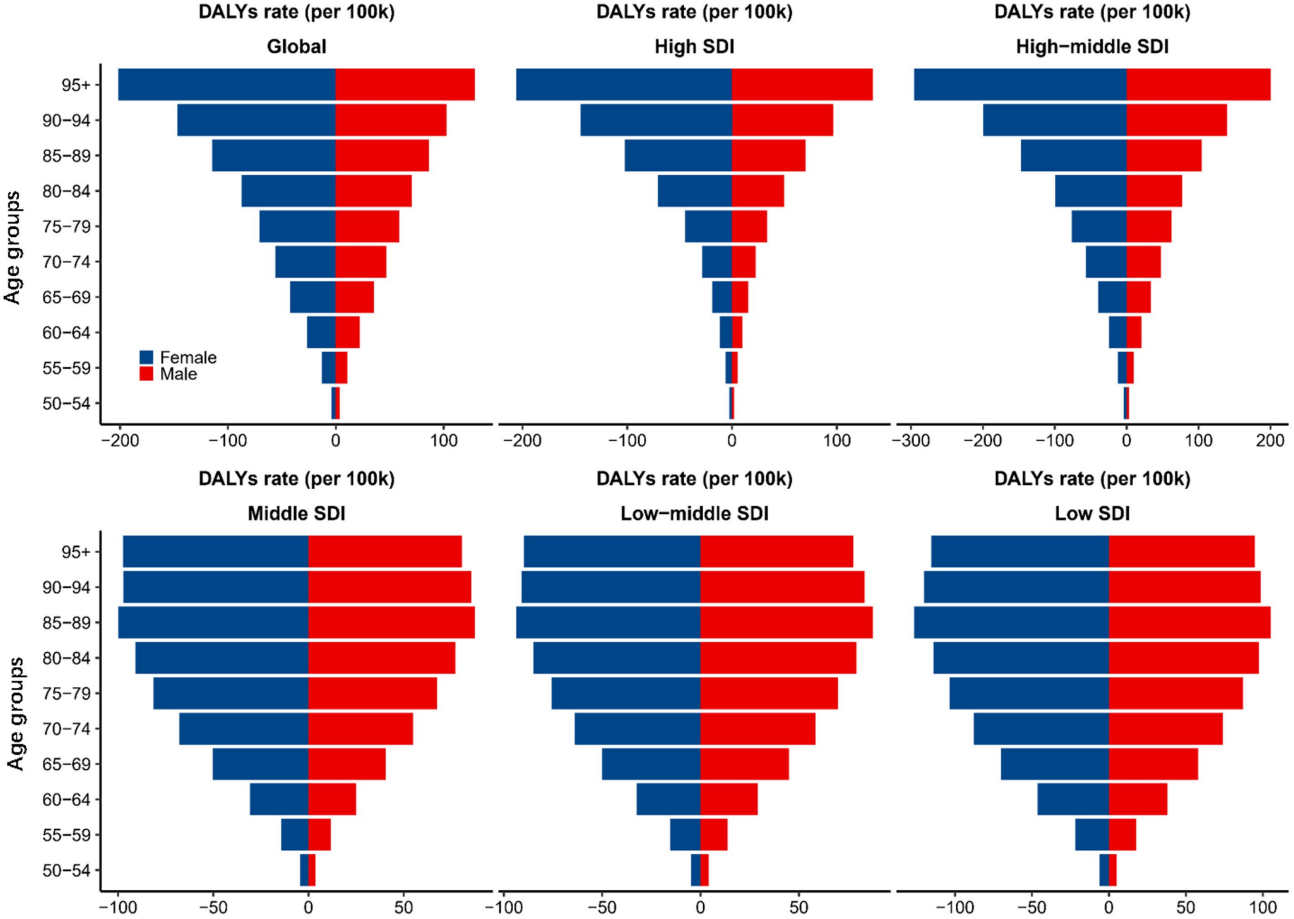
AMD DALYs rates (per 100,000) for 10 age groups globally and by SDI region, 1990–2019. AMD, age-related macular degeneration; DALYs, disability-adjusted life years; SDI, sociodemographic index.

The trends in the AMD DALY rates among the different age groups were also significantly different. As shown in Figure 2, globally, the AMD DALYs rate increased with age in both male and female. The same trend was found in the high- and high-middle-SDI regions. However, the AMD DALYs rates in the middle, low-middle, and low-SDI regions peaked in the 85–89 age group and then gradually declined with increasing age. In terms of sex disparities, in the high- and high-middle-SDI regions, there were no significant differences in the AMD DALYs rates between sexes in the 70–74 and younger age groups. However, a notable increase in burden for female compared to male was observed starting from the 75–79 age group. The remaining SDI regions had relatively smaller gender differences by age group.

### Age, period, and cohort effects on AMD DALYs

Globally, the net drift (annual percentage change) of AMD DALYs estimated by the APC model was −0.57% (95% CI: −0.72 to −0.43), with the highest net drift occurring in the low-middle-SDI region and the lowest occurring in the low-SDI region (Table 1). This indicates that the change in net drift is not necessarily consistent with the change in DALY number or age-standardized DALYs rate, which suggests that it is necessary to further analyse the period and cohort change trend of AMD DALYs.

According to the annual percentage change in AMD DALYs for each age group (local drift change in DALYs estimated by the APC model), we found that AMD DALYs decreased in all age groups, showing a horizontal “S”-shaped trend. The downwards trend weakened with age, with a turning point at 72.5 years, after which the decline increased with age again, with the greatest decline occurring at approximately 92.5 years (Supplementary Figure S1).

**Figure 3:**
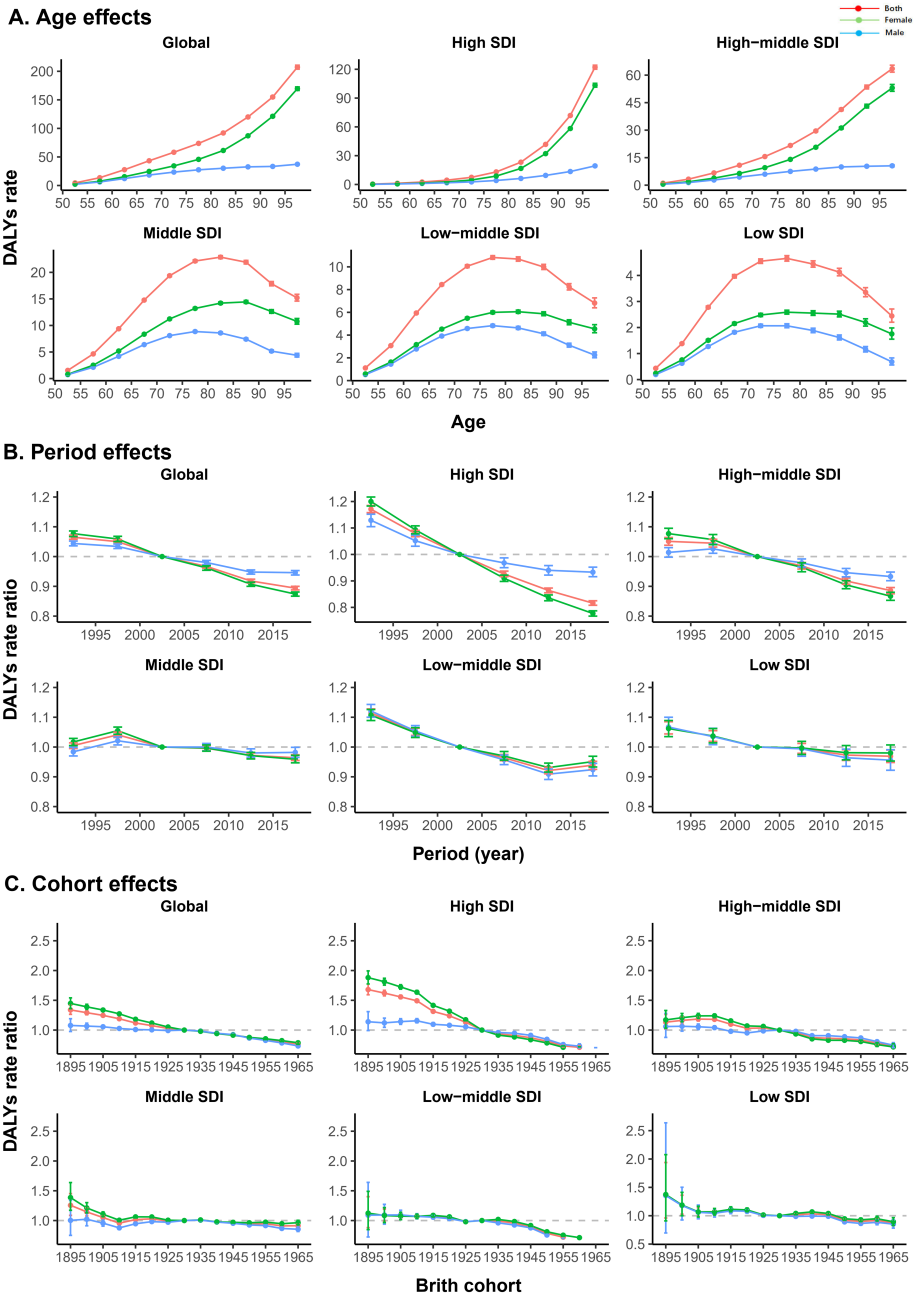
Age, period, and cohort effects on AMD DALYs by SDIs. **(A)** Age effects are represented by a longitudinal age curve of the DALYs rates (per 100,000) adjusted for period deviations. **(B)** Period effects are expressed by the relative risk of DALYs, and age-specific rates are calculated from 1990–1994 to 2015–2019 (reference period, 2000–2004). **(C)** Cohort effects are expressed as the relative risk of DALYs, and age-specific rates are calculated for the 1895 to 1965 cohort (reference cohort, 1930). The figure shows the DALYs rates and their corresponding 95% confidence intervals. AMD, age-related macular degeneration; DALYs, disability-adjusted life years; SDI, sociodemographic index.

As shown in Figure 3, the age effect also varied by SDI region. Generally, the burden of AMD increased with age in high- and high-middle-SDI regions, with the greatest burden occurring at 97.5 years. The low, low-middle, and middle SDI regions showed an inverted “U”-shaped trend, with peaks at 77.5 years, 77.5 years, and 82.5 years, respectively. With respect to the effect of age, for each SDI region, the change trend for male and female was consistent with the overall trend, but the burden of AMD in female in all age groups was significantly greater than that in male. In terms of the period effect, the burden of AMD generally decreased, except in the middle-SDI region. Among them, the high-SDI region showed the most significant decline during the period from 1990 to 2019. The low-SDI region showed little change in the period effect over the past two decades, indicating that the burden of AMD basically did not improve. However, the low-middle-SDI region showed a slight increase in the period effect in the past 10 years, indicating that the burden of disease may increase. The period effect in the middle-SDI region remained essentially unchanged from 1990 to 2019. In terms of sex, in the high- and high-middle-level regions, the reduction in the risk during the period effect was significantly greater for female than for male, but no significant sex differences were observed in the other SDI regions. Globally, the birth cohort effect revealed that the burden of AMD was lower in younger birth cohorts in general. Compared with other SDIs, the cohort effect was more pronounced in the high-SDI region, especially in the female group born between the 1890s and 1930s, where the decline in the burden of AMD was most significant. The burden of disease in the high-middle and low-middle SDI regions did not decrease notably until the birth cohorts after 1955 and 1945, respectively, and there was no significant sex difference.

### Age, period, and cohort effects in exemplary countries

**Figure 4:**
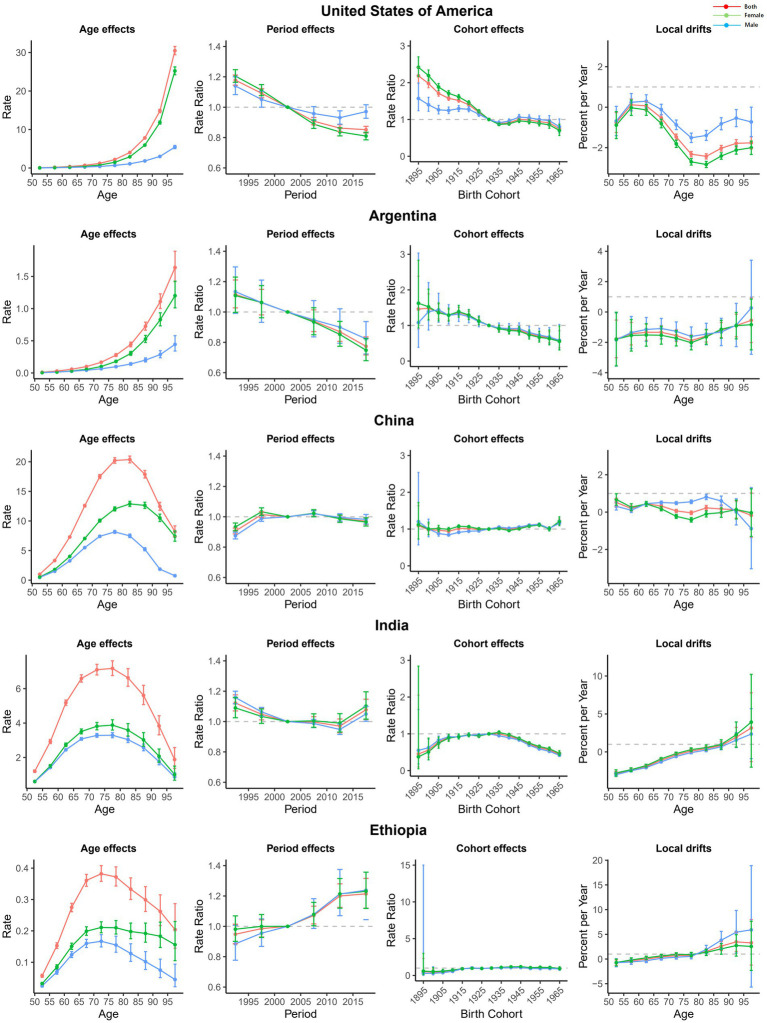
Age, period, and cohort effects on AMD DALYs in exemplary countries. AMD, Age-related macular degeneration; DALYs, disability-adjusted life years.

To more specifically describe the impact of APC on major trends in AMD worldwide, we selected an exemplary country for different SDI regions, as shown in Figure 4. In the United States, a typical representative high-SDI country, except for the 55–65 age group, the local drift of other age groups was less than 0, and the period effect showed a decreasing trend. The cohort effect was mainly reflected in the group before the 1930s, but there was no obvious change after that. Argentina, a high-middle-SDI country in South America, experienced a decline in local drift across all age groups and a marked declining trend in period and cohort effects. China is a middle-SDI country, with little change in age, period, or cohort effects, suggesting that the improvement in AMD disease burden in China was not significant from 1990 to 2019. India is the second most populous country in the world and is a low-middle-SDI country, and its AMD DALYs were gradually increasing in different age groups, especially after 75–80 years old. The period effect was also unfavorable. Compared with the 1930 cohort, the burden in the other cohorts was relatively lower. In Ethiopia, one of the low-SDI countries, the annual percentage change in AMD DALYs also increased with age, especially after 80 years. The influence of the period effect also increased year by year, but the cohort effect remained almost unchanged.

## Discussion

AMD is one of the major eye blindness diseases in the world and it is one of the public health problems that most countries have to face (1). The purpose of this study was to analyse the influence of global AMD burden variation, regional differences and age-period-cohort effects on disease burden, with the aim of providing an epidemiological basis and public health guidance for the formulation of global AMD prevention and control strategies. From 1990 to 2019, we found that the number of AMD DALYs increased by 90%, while the age-standardized DALYs rates decreased by approximately 15% worldwide. There were also differences in age-standardized DALYs rates across SDI regions and countries. The effects of age, period, and cohort on the burden of disease in AMD analysed by the APC model differed. At the same time, we also found that female in higher socioeconomic status countries had a greater burden of disease than male in all age groups.

In 1990–2019, the number of AMD DALYs almost doubled globally and across SDI regions. The massive growth in the global population is one of the main reasons. In addition, the aging of the population is also a major social problem that many countries face (20). According to statistics, the percentage of the world’s population aged 65 and over was 18% in 2000, while this number is expected to reach 38% by 2050 (21). This has led to a significant increase in the prevalence of AMD, which is a disease significantly associated with age. The burden of AMD in different SDI regions also varied greatly. We found that the number of DALYs was the highest in the middle-SDI region, followed by the high-middle-SDI region, and the lowest in the low-SDI region. There are two possible explanations for this phenomenon. First, China is a middle-SDI country, with the highest number of AMD DALYs in the world. Second, the high-middle SDI is mainly in some European countries with relatively high levels of social and economic development, and these countries are often faced with the problem of population aging caused by the extension of average life expectancy. However, further analysis of the age-standardized DALYs rates revealed that the burden of AMD was the lowest in the high-SDI region and the highest in the low-SDI region, especially in some African countries, where the annual percentage change in AMD DALYs even showed an increasing trend. This phenomenon was strongly related to the poor medical status. There is a severe shortage of human resources for health in Africa. Sub-Saharan Africa has a population of approximately 821 million people (more than Europe) but an estimated 145,000 doctors (only 5% of European practitioners) (22). Inadequate health budgets and poor leadership and management are also major challenges for healthcare systems in these regions (23). In addition, a study noted that the average number of years of education is negatively correlated with the age-standardized DALYs rates (24). Obviously, populations with higher levels of education tend to have greater awareness of health care.

The APC model was used to analyse the temporal trends in the global burden of AMD and determine the change in the burden of disease by period and birth cohort, thus allowing for an in-depth assessment of the effectiveness of relevant healthcare systems in the management of AMD. The age-standardized DALYs rates showed an inverse change from the number of DALYs mentioned above, and the net drift of AMD DALYs estimated by the APC model also decreased. This shows that humans have achieved some results in the fight against AMD. This can be attributed mainly to the following aspects. First, the rapid development of medical technology and related examination equipment. The clinical application of laser scanning ophthalmoscopy (SLO) and optical coherence tomography (OCT) has greatly improved the diagnosis rate for AMD (25, 26). The second is the remarkable clinical effect of anti-vascular endothelial growth factor (27). The third is global efforts (28). These factors have all contributed to improvements in the burden of AMD in most parts of the world. However, as mentioned above, due to global population growth and aging, the prevalence and vision burden of AMD will remain a difficult challenge in the coming years.

Analysis of the APC model revealed that, in terms of age effects, the global burden of disease was mainly attributable to older populations in high- and high-middle-SDI regions. Age is an important risk factor for the development of AMD. With increasing age, the high-middle and high-SDI regions showed similar “exponential” changes, among which the 97.5-year-old group had the heaviest burden. In the middle, low-middle, and low-SDI regions, the age of the heaviest burden group was relatively younger, mainly at 75–85 years. There are two possible explanations for this phenomenon. First, countries or regions with higher SDIs have better health care systems and medical conditions and higher levels of population awareness, which enables early detection and treatment of AMD to delay the progression of the disease, thus greatly reducing the burden on younger age groups. However, for lower-SDI regions, because early AMD often has little impact on vision, patients are not easy to pay attention to, and because of the lack of diagnosis and treatment, early intervention is often unavailable. Therefore, it shifts the burden of disease to the younger group. Second, the difference in life expectancy and composition of the population also affects the variation in burden across age groups. Countries with lower SDIs tend to have shorter average life expectancies and a relatively lower proportion of older people. For example, the average life expectancy of India, a low-middle-SDI country, is only 68.3 years, while the average life expectancy of Japan, a high-SDI country with the most aging population, is 83.7 years (29). As a result, older age groups in developed countries often have a heavier burden of disease.

By analysing the period effects, we found that the trends were most significant in the high- and high-middle-SDI regions, where the burden of AMD significantly decreased. Obviously, higher socioeconomic levels can reduce the burden of disease more because more developed countries can not only provide better medical care services but also have more access to a variety of ophthalmic precision instruments and cutting-edge medicines. In 1982, Mainster et al. first applied SLO to clinical ophthalmology (25), and by the 1990s, the application and continuous innovation of OCT technology (26, 30) in ophthalmology has made the diagnosis of AMD new. At the same time, anti-VEGF agents were approved for marketing in 2006; these agents not only significantly improved the visual function of patients but also completely changed the treatment strategy for AMD (31). However, for countries or regions with low levels of economic development, national public health fiscal spending is limited, most hospitals do not have the ability to equip advanced medical equipment, and most patients cannot afford expensive medicines. On average, nearly 50% of health care financing in low-income countries comes from out-of-pocket payments, compared with 30% in middle-income countries and only 14% in high-income countries (32). As a result, low-SDI countries may not be well positioned to obtain the benefits of sophisticated instrumentation and cutting-edge medicines.

The cohort effect was mainly reflected in the female in the pre-1930 cohort in the high-SDI region, which mainly showed that the earlier the birth cohort was, the greater the disease burden. Before the 19th century, female’s social status was very low, social medical resources were usually biased towards male, and female were often treated unequally in the medical field (33). However, around the 20th century, female in some countries with higher socioeconomic levels awakened their consciousness and began to fight for their rights and interests (34). Although female’s social status and rights were still greatly restricted, medical and research institutions focusing on female’s health have begun to emerge in some countries to provide better medical services and health security for female. Female were gradually allowed to study medical knowledge (35). This may explain why the pre-1930 cohort of female in the high-SDI region experienced the most pronounced changes in the burden of AMD.

According to the APC model, we also found that there was a significant sex difference in the burden of AMD, with a higher rate of AMD DALYs in female than in male across all age groups. Notably, this phenomenon was more pronounced in SDI regions with higher socioeconomic levels than in regions with lower socioeconomic levels. Similarly, previous studies have reported a greater incidence and heavier burden of AMD in female (36, 37). This may be strongly related to the longer life expectancy and greater proportion of females in the older population. According to the official website of the World Health Organization (WHO), in 2020, the average life expectancy of female in high-income countries was generally greater than that of male. However, in low-income countries, the average life expectancy is only approximately 65 years, and the limited access to care resources for both male and female may result in a lack of a significant sex difference in the burden of disease in low-income countries. In other words, the longevity of female in countries with high levels of economic development also increases the sex difference in AMD to some extent. At the same time, female may be more susceptible to AMD for physiological reasons. Some studies have proposed that the occurrence and development of AMD may be related to factors such as estrogen, the number of X-linked genes, and lactation (38–40), but there is still a lack of strong evidence to support these views. In addition, there may be gender inequality in the diagnosis and treatment of diseases. Female’s health needs are more likely to be overlooked due to their different social roles (41). A Swedish study revealed that female patients often had worse vision before ophthalmologic surgery and had longer waiting times for surgery than male patients (42). Inequities in various social factors, such as income level, employment opportunities, financial decision-making power, education level, disease awareness, and personal freedom, may contribute to gender differences in disease burden. Focusing on female’s eye care services is also an important component of addressing the global burden of AMD. Therefore, more attention should be given to older female to obtain a more targeted and effective approach to prevention and treatment.

The APC model also allows for the comparison of trends across countries. The USA has the most advanced medical technology worldwide, but AMD is still an important cause of blindness for Americans. According to statistics, 18.34 million people aged 40 and above in the USA have early AMD, with a crude prevalence of 11.64% (43). The burden of disease among older Americans is still severe. China is the most populous country worldwide and the country with the heaviest burden of AMD. According to the White Paper on Eye Health in China released in 2020, there are only 1.6 ophthalmologists per 50,000 people in China, most of whom are concentrated in urban hospitals at the prefecture level and above, and medical resources in rural and remote areas are seriously lacking. India spends only 1.04% of gross domestic product (GDP) on public health, including private sector contributions, and only 18% of the urban poor are covered by health insurance (44). For countries with large populations, the medical system and social security system should be further improved, which is highly important for reducing the global burden of AMD. At present, China has formulated several plans to prevent and treat blindness. The “14th Five-Year” National Eye Health Plan (2021–2025) lists the prevention and treatment of AMD as one of the important tasks and specifies goals such as strengthening the construction of the ophthalmology medical service system, improving the capacity of ophthalmology medical services, and optimizing ophthalmology professional and technical personnel. Most African countries, such as Ethiopia and Côte d’Ivoire, have severe burdens of AMD, which may be related to population growth, low standards of living, and poor health. The average life expectancy of Africans is only 54 years, which is much lower than the global average (45). The health sector in most African countries is underfunded. In Nigeria, public health expenditures were only 5.8% of GDP in 2009 (46). For African countries, improving basic health and medical care is particularly important.

Vision impairment caused by AMD is a major problem for humans, and to improve and protect vision health, many countries around the world have developed corresponding eye care goals, policies and programs. Although the planning time of the “VISION 2020” has passed and the goal has not been fully achieved, it is a landmark global eye health program. In 2013, the WHO further proposed the Global Action Plan for Eye Health (GAP), which is a wide-ranging and ongoing program supported by many countries. The GAP aims to reduce vision loss and blindness globally by strengthening eye health services, promoting research and innovation, enhancing public awareness and education, and strengthening international cooperation and exchange. These eye health plans express the firm commitment and concrete action direction around the world to improve eye health, which is not only to reduce human vision loss but also to achieve sustainable development.

This study also has some limitations. First, the GBD database was based on a wide range of data sources, and the DALYs data were mainly estimated by representative stratified models based on population-based studies; therefore, the data may be incomplete or biased due to factors such as region, time, and population. Second, the GBD database used the International Classification of Diseases as the basis for disease classification. However, different countries and regions are affected by technological and resource constraints, which may lead to inaccurate or missing AMD diagnoses, thus widening the differences in the burden of disease among regions. Third, the GBD 2019 study did not include data on other epidemiologic factors such as genetic factors, lifestyle habits, environmental factors, racial factors, and systemic diseases in AMD, but this information is also important to guide global disease prevention and control efforts.

In order to effectively reduce the burden on patients, we advocate that future research should focus on in-depth analysis of the burden of disease in specific countries, so as to propose more precise and efficient disease prevention, control and treatment programs. In addition, given the limitations of this paper, we encourage follow-up studies to fully integrate and utilize the resources of multiple authoritative databases and implement a multifactorial and multidimensional analysis strategy, aiming to more comprehensively explain the influences and interactive effects of a variety of AMD-related factors in epidemiology, thereby enriching AMD prevention and treatment strategies.

## Conclusion

This study analysed changes and differences in the burden of AMD globally and across regions from 1990 to 2019 and explored the effects of age, period, and cohort on AMD DALYs. Although the age-standardized DALYs rate in AMD patients has declined in most countries, there has still been a significant increase in the number of DALYs due to the growth and aging of the population. The loss of vision associated with AMD remains a difficult challenge. More efforts still need to be made by countries worldwide in prevention and control measures.

## Data Availability

Publicly available datasets were analyzed in this study. This data can be found at: https://vizhub.healthdata.org/gbd-results/.
