# Supplementary material for: Global, regional, and national burden of age-related macular degeneration, 1990–2019: an age-period-cohort analysis based on the Global Burden of Disease 2019 Study
Source: Front Public Health. 2024 Oct 22;12:1486168. doi: 10.3389/fpubh.2024.1486168 (PMC11534605; doi:10.3389/fpubh.2024.1486168)
Supplement: Supplementary file 2 [file Image_1.PDF]

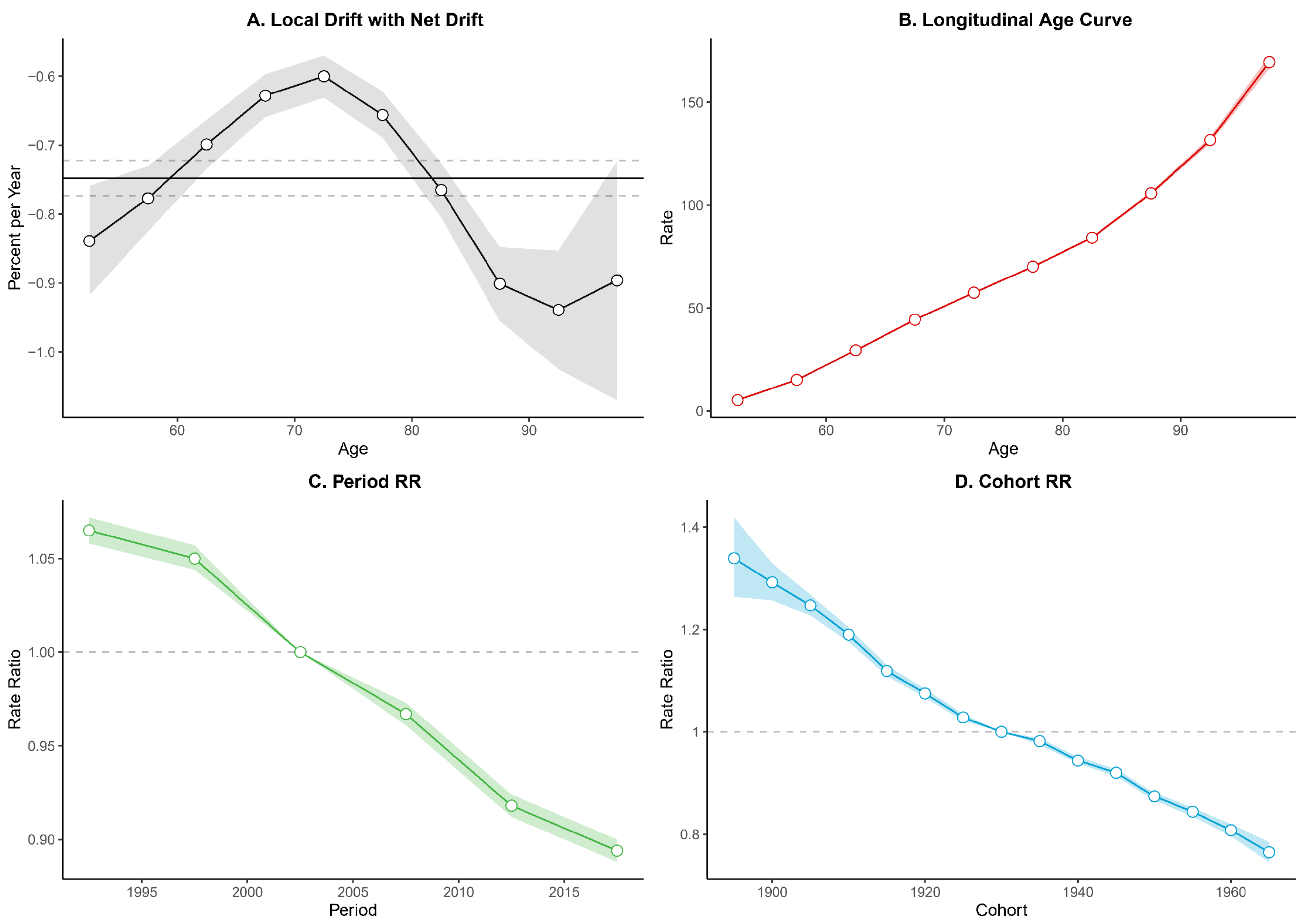

A. Local Drift with Net Drift
B. Longitudinal Age Curve
C. Period RR
D. Cohort RR
Percent per Year
−0.6
−0.7
−0.8
−0.9
−1.0
Age
60
70
80
90
Rate
150
100
50
0
Rate Ratio
1.05
1.00
0.95
0.90
Period
1995
2000
2005
2010
2015
1.4
1.2
1
0.8
Cohort
1900
1920
1940
1960

Supplementary Figure 1. Age, period, and cohort effects on AMD DALYs. **(A)** Local drift of AMD DALYs in 10 age groups (50-54 years to 95+ years), 1990 to 2019. **(B)** Age effects are represented by a longitudinal age curve of the DALYs rates (per 100,000) adjusted for period deviations. **(C)** Period effects are expressed by the relative risk of DALYs, and age-specific rates are calculated from 1990-1994 to 2015-2019 (reference period, 2000-2004). **(D)** Cohort effects are expressed as the relative risk of DALYs, and age-specific rates are calculated for the 1895 to 1965 cohort (reference cohort, 1930). The dots and shaded areas represent the DALYs rates and the corresponding 95% confidence intervals, respectively. AMD: age-related macular degeneration, DALYs: disability-adjusted life years.
